# Male and Female Adult Population Health Status in China: A Cross-Sectional National Survey

**DOI:** 10.1186/1471-2458-8-277

**Published:** 2008-08-05

**Authors:** Jing Shi, Meina Liu, Qiuju Zhang, Mingshan Lu, Hude Quan

**Affiliations:** 1 Division of Preventive Medicine, Department of Basic Medical Science, Hebei Medical University, Shijiazhuang, PR China; 2 Department of Biostatistics, School of Public Health, Harbin Medical University, Harbin, PR China; 3 Department of Economics, University of Calgary, Calgary, Canada; 4 Department of Community Health Sciences and Centre for Health and Policy Studies, University of Calgary, Calgary, Canada

## Abstract

**Background:**

With rapid economic growth and globalization, lifestyle in China has been changing dramatically. This study aimed to describe the male and female adult Chinese population health status.

**Methods:**

The Chinese Third National Health Services Survey was conducted in 2003 to collect information about health status and quality of life from randomly selected residents. Of the 193,689 respondents to the survey (response rate 77.8%), 139,831 (69,748 male and 70,083 female) respondents who were 18 years of age or older were analyzed.

**Results:**

Among the respondents, fewer males than females rated their overall wellbeing as being poor or very poor (4.8% versus 6.2%), reported illness in the last 2 weeks (14.1% versus 17.4%), presence of physician diagnosed chronic disease (15.0% versus 17.7%) and at least one functional problem in seven items of the quality of life (26.9% versus 32.8%). More males than females were currently smoking (52.4% versus 3.4%) and drank alcohol more than three times per week (16.5% versus 1.1%). Physically inactive rate was similar between males and females (85.8% versus 87.0%). Fewer rural respondents reported chronic disease than urban respondents (13.0% versus 19.9% for males and 15.5% versus 22.8% for females). In all seven items of the quality of life measured, rural respondents reported less problems than urban respondents (26.2% versus 28.7% for males and 32.0% versus 34.7% for females).

**Conclusion:**

Males had better health status than females in terms of self-perceived wellbeing, presence of illness, chronic disease, and quality of life. However, smoking and frequent alcohol drinking was more prevalent among males than that among females. In contrast with the social-economic gradient in health commonly found in the literature, the wealthier urban population in China was not found to be healthier than the rural population in terms of physician diagnosed chronic disease.

## Background

China's population of 1.3 billion accounts for 20% of the world population, making it the most populated country in the world. With rapid economic growth and globalization, Chinese people's lifestyle has been changing dramatically towards being more physically inactive, eating more fast food, and being overall more stressed compared their old lifestyle. Because of the rapid change in lifestyle, population health is quickly shifting from a high mortality rate due to infectious and domestic diseases, related to 'poverty' before implementation of 'open door policy' in 1980, to a currently greater life expectancy and higher prevalence of chronic and non-communicable diseases brought on by 'affluence' [1].

In the last five decades, the Chinese population has become healthier as measured by decreased mortality rate and increased life expectancy. For example, infant mortality has decreased from 80.8/1000 live births in 1958 to 21.5/1000 live births in 2004 [2], maternal mortality has declined from 88.9/100000 live births in 1990 to 48.3/100000 live births in 2004, and life expectancy has increased from 57.0 years in 1957 to 71.8 years in 2004 [3]. The most recent data showed that heart disease, cancer and stroke are the major causes of death, accounting for 65% of all deaths and infectious disease has become the least important cause of death, only accounting for 3% of all deaths in Chinese population aged 40 or above [4].

Life expectancy is an internationally used health measure because many countries, including China, periodically calculated it using vital data. However, such measure mirrors mortality, and particularly infant mortality, but is insensitive to nonfatal and psychosocial conditions that contribute indirectly to death. Hence, it is imperative to assess population health using indicators that reflect contemporary health issues. This study aimed to describe the male and female adult Chinese population health status in multiple dimensions, including well-being, morbidity, quality of life, and health behaviors of smoking, alcohol consumption and physical activity, using data from the most recent National Health Services Survey from the Chinese government. Our descriptive study on Chinese population health status has application for international organizations because China's large population will influence global health status. Our study provided important information for generating research questions for future studies.

## Methods

### Study Population

We derived data from the China Third National Health Services Survey, which collected data through face-to-face interviews from September 18 to October 20, 2003. Of the 193,689 respondents surveyed, we included individuals who were 18 years of age or older and excluded 3,677 respondents with missing values, resulting in a total of 139,831 (69,748 males and 70,083 females) respondents in our analysis.

The national survey employed a multiple stage cluster sampling method to select the sample randomly. The mainland of China was clustered according to the government administrative geographic system (i.e., county, town and village in rural areas, and city, community, and neighbourhood in urban areas). Firstly, 95 counties and cities were randomly selected from rural and urban areas. Secondly, 5 towns and 5 communities were randomly selected in each county and city, respectively. Thirdly, 2 villages in each town and 2 neighbourhoods in each community were randomly selected. Fourthly, 60 households were randomly selected in each village and neighbourhood, respectively, resulting in about 57,000 households. All family members aged 15 years or older were invited to participate in the face-to-face interview.

### Data collection

Medical doctors and nurses conducted the survey. Before the survey, interviewers were trained and practiced interviewing; their understanding and knowledge about the survey method and content were examined through testing. During the survey, interviewers visited each household up to three times on different days and times. Interviewers explained the purposes and confidentiality of the survey, and then invited family members to participate. Respondents could choose not to participate and their participation in the survey was accepted as oral consent. The completeness of questionnaires was checked by a district survey manager at the end of every day. If there was missing information on the survey, individuals would be re-surveyed if possible. After the survey, 5% of households were randomly selected and re-surveyed on 14 questions to examine survey quality; the agreement was 95%. The survey response rate for adults was 77.8% [5].

### Demographic Characteristics

Demographic variables included age, sex, marital status, education; rural/urban residence, and geographic region. Educational level was categorized into five categories, illiterate (it was defined as people who could not read newspaper or magazines, or write a short note), elementary school (i.e. those who attended up to 6 years of schooling or were not illiterate for those without schooling), junior high school (i.e. schooling 7 – 9 years), senior high school (i.e. schooling 10–12 years), and college or university or higher (i.e. complete or incomplete of post-secondary school). Residence was divided based on rural and urban area and then economic development. Rural area included towns and villages. Based on economic development, Eastern China, the most developed region, included 11 provinces and metropolitans such as Beijing, Shanghai, and Liaoning. Middle China included 8 provinces, such as provinces of Heilongjiang, Shanxi, and Hunan. Western China, the least developed region, included 12 provinces such as Yunnan, Tibet, In-Mongolia, and Ningxia.

### Health Status Indicators

Self-perceived overall wellbeing was assessed using a five -point Likert-type scale of being excellent, good, fair, poor or very poor. Presence of illness in the last two weeks and physician-diagnosed chronic disease in the last six months was recorded. The two-week illness was surveyed by asking: "Have you had any physical and mental discomforts during the last two weeks?" Chronic disease referred to disease diagnosed by medical doctors and occurring in the last 6 months prior to the survey, or chronic disease that was diagnosed more than 6 months prior to the survey but reoccurred within the last 6 months and received treatment. Non-physician diagnosed chronic disease was not included because the validity of self-diagnosed medical conditions depends on the level of the respondent's knowledge and their perceptions on the definition of 'disease' and 'health'. Physician diagnosed chronic disease was further confirmed by asking diagnosis location including community clinics, county hospital, city hospital, provincial hospital, military hospital, and others. Respondent reported up to three specific chronic diseases. The reported diseases were coded and classified using the disease classification scheme designed by China Ministry of Health for the survey.

Quality of life was measured using a seven-item instrument. Respondents were asked about presence and level of severity of their dysfunction and disability in the last 30 days in 1) ability about washing or dressing themselves, 2) ability to do job work or housework, 3) feeling of pain or physical discomfort, 4) ability of concentration on work or study and memory, 5) ability of recognizing familiar people from 20 meters away (with glasses for those wearing glasses), 6) emotional discomfort due to restlessness, and 7) anxiety or depression. Under each item, five itemized answers about presence and severity were provided, including: none, mild, moderate, severe, and extremely severe.

### Health determinant

Information about smoking, alcohol consumption and physical exercise was collected. For smoking, the survey asked: "Are you currently smoking?" (with answer: Yes, No) Under the survey question of "Do you drink alcohol?", the three answers were provided: "No or rarely", "Sometimes" (defined drinking < 3 times per week), and "Frequently" (defined drinking ≥ 3 times per week). For exercise, a question of "What is the sport or exercise that you have been regularly doing in the last 6 months?" was asked with providing a list of recreational physical activities, such as running, Tai Chi, Wushu, dancing, and playing balls. Regularity of exercise was not defined in the survey and determined by respondent's perception.

### Statistical Analysis

Proportion was employed to describe respondents in demographic characteristics, health status and health determinants. Because of the large sample size and multiple categories in some variables, the P-value for sex difference was not reported. Frequencies of variables in the survey were not weighted because sampling weight was not available. The same sampling method had been used in the previous two National Health Services Surveys in China. Analyses of previous surveys suggest that this sampling method is adequate to generate a nationally representative sample [5]. The survey respondent age and sex composition was comparable with the 2000 census. Finally, multiple logistic regressions were used to generate risk adjusted P-value for gender difference in health indicators after adjustment for demographic characteristics and correction of clustering of individuals within family using the repeated measure [6,7].

The data were analyzed at the health information centre of the Ministry of Health in Beijing. Confidentiality of the survey was protected through storing the data on password protected computers at the Ministry, and removing personal identifiable information (such as name and address) from the database available for researchers and examining analysis outputs for release of aggregated data by the centre staff.

## Results

### Demographic Characteristics

Demographic characteristics for respondents are presented in Table 1. A majority of the respondents were married (80.3%) and resided in rural areas (71.2%). There was a similar proportion of male and female (49.9%. versus 50.1%). More males than females were unmarried (14.8% versus 9.2%) but had higher education (illiterate rate: 12.2% for males and 27.7% for females). The composition by age, rural/urban and region was similar between males and females.

**Table 1:** Characteristics of the survey respondents aged 18 years or older in China

Variables	Total N (% of 139831)	Male N (% of 69748)	Female N (% of 70083)
Age			
18–34	43055 (30.8)	21475 (30.8)	21580 (30.8)
35–44	31770 (22.7)	15646 (22.5)	16124 (23.0)
45–54	30023 (21.5)	15088 (21.6)	14935 (21.3)
55–64	16942 (12.1)	8746 (12.5)	8196 (11.7)
≥ 65	18041 (12.9)	8793 (12.6)	9248 (13.2)
Marital status			
Married	112274 (80.3)	55622 (79.8)	56652 (80.8)
Unmarried	16736 (12.0)	10324 (14.8)	6412 (9.2)
Divorce	1584 (1.1)	938 (1.3)	646 (0.9)
Widow	9237 (6.6)	2864 (4.1)	6373 (9.1)
Education			
Illiterate	27905 (20.0)	8494 (12.2)	19411 (27.7)
Elementary school	38332 (27.4)	19374 (27.7)	18958 (27.1)
Junior high school	45654 (32.7)	25788 (37.0)	19866 (28.3)
Senior high school	15024 (10.7)	8787 (12.6)	6237 (8.9)
College or university	12916 (9.2)	7305 (10.5)	5611 (8.0)
Residence area			
Urban	40244 (28.8)	19516 (28.0)	20728 (29.6)
Rural	99587 (71.2)	50232 (72.0)	49355 (70.4)
Region of China			
East of China	48554 (34.7)	23957 (34.4)	24597 (35.1)
Middle of China	39056 (27.9)	19541 (28.0)	19515 (27.8)
West of China	52221 (37.4)	26250 (37.6)	25971 (37.1)

### Health Status and Determinant

Of the respondents, 5.5% rated their overall wellbeing as being poor or very poor, 15.8% reported illness in the last 2 weeks and 16.3% reported presence of chronic disease (see Table 2). Compared to males, more females rated their overall wellbeing as being poor or poorer (4.8% versus 6.2%, risk adjusted P < 0.001), and reported presence of illness in the last 2 weeks (14.1% versus 17.4%, risk adjusted P < 0.001) and chronic disease (15.0% versus 17.7%, risk adjusted P < 0.001). Males had lower prevalence of heart disease (1.4% versus 2.4%), hypertension (3.2% versus 4.0%) and rheumatologic arthritis (0.8% versus 1.5%) than females. However, prevalence of chronic pulmonary disease was slightly higher for males than that for females (1.7% versus 1.1%).

**Table 2:** Self-perceived overall physical and emotional wellbeing, illness, and morbidity in the respondents aged 18 years or older in China

	Total N (% of 139831)	Male N (% of 69748)	Female N (% of 70083)
*Physical and emotional wellbeing*			
Excellent	49088 (35.1)	25816 (37.0)	23272 (33.2)
Good	50996 (36.5)	26156 (37.5)	24840 (35.5)
Fair	32055 (22.9)	14447 (20.7)	17608 (25.1)
Poor	6724 (4.8)	2873 (4.1)	3851 (5.5)
Very poor	968 (0.7)	456 (0.7)	512 (0.7)
Combination of poor and very poor*	7692 (5.5)	3329 (4.8)	4363 (6.2)
*Morbidity*			
Presence of illness in the last 2 weeks before the survey*	22050 (15.8)	9865 (14.1)	12185 (17.4)
Presence of physician diagnosed chronic disease in the last 6 months before the survey*	22808 (16.3)	10432 (15.0)	12376 (17.7)
Infectious and parasitic disease	485 (0.4)	303 (0.4)	182 (0.3)
Cancer	231 (0.2)	122 (0.2)	109 (0.2)
Diabetes	1062 (0.8)	472 (0.7)	590 (0.8)
Heart disease*	2644 (1.9)	997 (1.4)	1647 (2.4)
Stroke	1257 (0.9)	693 (1.0)	564 (0.8)
Chronic pulmonary disease*	1911 (1.4)	1153 (1.7)	758 (1.1)
Hypertension*	4989 (3.6)	2203 (3.2)	2786 (4.0)
Peptic ulcer	707 (0.5)	446 (0.6)	261 (0.4)
Chronic liver disease	192 (0.1)	123 (0.2)	69 (0.1)
Chronic renal disease	258 (0.2)	83 (0.1)	175 (0.3)
Rheumatologic arthritis*	1598 (1.1)	534 (0.8)	1064 (1.5)

* Note: P value < 0.001 for males versus females after adjustment for age, marital status, education, urban/rural residence and geographic region.

In all seven items of the quality of life, 29.9% reported at least one problem (see Table 3). The rate was significantly lower for males than that for females (26.9% versus 32.8%, risk adjusted P < 0.001). Males were more likely than females to report no problems on all seven items (such as 86.6% versus 81.8% for pain, 88.1% versus 84.3% for concentration or memory, 90.6% versus 87.5% for vision, 88.2% versus 84.3% for emotional discomfort and 89.3% versus 86.1% for anxiety/depression).

**Table 3:** Quality of life in the respondents aged 18 years or older in China

Quality of life	Total N (% of 139831)	Male N (% of 69748)	Female N (% of 70083)
*Problem about washing or dressing yourself in the last 30 days*			
No problem	131927 (94.4)	66277 (95.0)	65650 (93.7)
Mild problem	5193 (3.6)	2216 (3.1)	2977 (4.2)
Moderate problem	1666 (1.2)	734 (1.1)	932 (1.3)
Severe problem	777 (0.6)	389 (0.6)	388 (0.6)
Extremely severe problem	268 (0.2)	132 (0.2)	136 (0.2)
*Problem about usual activities such as work, or housework in the last 30 days*			
No problem	126403 (90.4)	64061 (91.9)	62342 (89.0)
Mild problem	8876 (6.3)	3621 (5.2)	5255 (7.5)
Moderate problem	2723 (2.0)	1186 (1.7)	1537 (2.2)
Severe problem	1362 (1.0)	644 (0.9)	718 (1.0)
Extremely severe problem	467 (0. 3)	236 (0.3)	231 (0.3)
*Level of pain and physical discomfort in the last 30 days*			
No pain or physical discomfort	117701 (84.2)	60364 (86.6)	57337 (81.8)
Mild pain or physical discomfort	16228 (11.6)	6835 (9.7)	9393 (13.4)
Moderate pain or physical discomfort	4373 (3.1)	1847 (2.7)	2526 (3.6)
Severe pain or physical discomfort	1274 (0.9)	580 (0.8)	694 (1.0)
Extremely severe pain or physical discomfort	255 (0.2)	122 (0.2)	133 (0.2)
*Problem about concentration or memory in the last 30 days*			
No problem	120534 (86.2)	61433 (88.1)	59101 (84.3)
Mild problem	14232 (10.2)	6173 (8.8)	8059 (11.5)
Moderate problem	3765 (2.7)	1553 (2.2)	2212 (3.2)
Severe problem	1027 (0.7)	467 (0.7)	560 (0.8)
Extremely severe problem	273 (0.2)	122 (0.2)	151 (0.2)
*Problem of recognizing a familiar person in 20 meter or more away (with glasses for people wearing glasses)*			
No problem	124449 (89.0)	63157 (90.6)	61292 (87.5)
Mild problem	9895 (7.1)	4354 (6.1)	5541 (7.9)
Moderate problem	3772 (2.7)	1503 (2.2)	2269 (3.2)
Severe problem	1316 (0.9)	558 (0.8)	758 (1.1)
Extreme problem	399 (0.3)	176 (0.3)	223 (0.3)
*Problem about emotional discomfort due to restlessness in the last 30 days*			
No problem	120632 (86.3)	61542 (88.2)	59090 (84.3)
Mild problem	15504 (11.1)	6680 (9.6)	8824 (12.6)
Moderate problem	2938 (2.1)	1185 (1.7)	1753 (2.5)
Severe problem	586 (0.4)	258 (0.4)	328 (0.5)
Extremely severe problem	171 (0.1)	83 (0.1)	88 (0.1)
*Anxiety or depression in the last 30 days*			
No anxiety or depression	122612 (87.7)	62263 (89.3)	60349 (86.1)
Mild anxiety or depression	13230 (9.4)	5786 (8.3)	7444 (10.6)
Moderate anxiety or depression	3040 (2.2)	1271 (1.8)	1769 (2.5)
Severe anxiety or depression	764 (0.6)	349 (0.5)	415 (0.6)
Extremely severe anxiety or depression	185 (0.1)	79 (0.1)	106 (0.2)
Presence of mild, moderate, severe or extremely severe problem on any one of seven items above*	41756 (29.9)	18753 (26.9)	23003 (32.8)

* Note: P value < 0.001 for males versus females after adjustment for age, marital status, education, urban/rural residence and geographic region.

Respondents aged 65 years or older had much poorer health status than those aged less than 65 years old among males and females (see Table 4). A similar proportion of rural and urban respondents rated their health status as being poor or very poor (4.8% versus 4.7% for males and 6.3% versus 6.1% for females), and reported the presence of illness in the last 2 weeks (14.2% versus 14.0% for males and 17.3% versus 17.6% for females). However, fewer rural respondents reported chronic disease than urban respondents (13.0% versus 19.9% for males and 15.5% versus 22.8% for females). In all seven items of the quality of life measured, rural respondents reported fewer problems than urban respondents (26.2% versus 28.7% for males and 32.0% versus 34.7% for females).

**Table 4:** Self-perceived overall physical and emotional wellbeing, illness, morbidity and quality of life by age and region in the male and female respondents aged 18 years or older in China

	Male Age		Female Age		Male Residence		Female Residence	
				
Quality of life	< 65 N (%)	≥ 65 N (%)	< 65 N (%)	≥ 65 N (%)	Rural N (%)	Urban N (%)	Rural N (%)	Urban N (%)
Total N (denominator)	60955	8793	60835	9248	50232	19516	49355	20728
Perceived poor or very poor physical and emotional wellbeing	1905 (3.1)	1424 (16.2)	2589 (4.3)	1774 (19.2)	2419 (4.8)	910 (4.7)	3106 (6.3)	1257 (6.1)
Presence of illness in the last 2 weeks before the survey	7198 (11.8)	2667 (30.3)	9194 (15.1)	2991 (32.3)	7142 (14.2)	2723 (14.0)	8533 (17.3)	3652 (17.6)
Presence of physician diagnosed chronic disease in the last 6 months before the survey	6899 (11.3)	3533 (40.2)	8575 (14.1)	3801 (41.1)	6541 (13.0)	3891 (19.9)	7659 (15.5)	4717 (22.8)
Presence of mild to extremely severe problem in the last 30 days on any one of seven items below	13173 (21.6)	5580 (63.5)	16376 (26.9)	6627 (71.7)	13144 (26.2)	5609 (28.7)	15814 (32.0)	7189 (34.7)
Washing or dressing yourself	1815 (3.0)	1656 (18.8)	2322 (3.8)	2111 (22.8)	2559 (5.1)	912 (4.7)	3291 (6.7)	1142 (5.5)
Usual activities such as work, or housework	3113 (5.1)	2574 (29.3)	4320 (7.1)	3421 (37.0)	4179 (8.3)	1508 (7.7)	5584 (11.3)	2157 (10.4)
Pain and physical discomfort	6219 (10.2)	3165 (36.0)	8688 (14.3)	4058 (43.9)	6689 (13.3)	2695 (13.8)	8880 (18.0)	3866 (18.7)
Concentration or memory	4720 (7.7)	3595 (40.9)	6525 (10.7)	4457 (48.2)	5723 (11.4)	2592 (13.3)	7360 (14.9)	3622 (17.5)
Recognizing a familiar person in 20 meter or more away (with glasses for people wearing glasses)	3248 (5.3)	3343 (38.0)	4352 (7.2)	4439 (48.0)	4634 (9.2)	1957 (10.0)	5973 (12.1)	2818 (13.6)
Emotional discomfort due to restlessness	5531 (9.1)	2675 (30.4)	7525 (12.4)	3468 (37.5)	5668 (11.3)	2538 (13.0)	7479 (15.2)	3514 (17.0)
Anxiety or depression	5476 (9.0)	2009 (22.9)	7171 (11.8)	2563 (27.7)	5260 (10.5)	2225 (11.4)	6845 (13.9)	2889 (13.9)

Of the respondents, 27.9% were smoking, 8.8% drank alcohol frequently and 13.6% exercised regularly (see Table 5). Compared to males, females were significantly less likely to smoke (52.4% versus 3.4%, risk adjusted P < 0.001), drink alcohol (frequent alcohol consumption 16.5% versus 1.1%, risk adjusted P < 0.001) but less likely to do regular exercise (14.2% versus 13.0%, risk adjusted P < 0.001). Smoking and frequent alcohol consumption rate were particularly high among males aged 35 to 64 years and regular exercise rate was especially high among male and female seniors (age ≥ 65 years) and among urban respondents (see Table 6).

**Table 5:** Prevalence of smoking, alcohol consumption and physical activity in the respondents aged 18 years or older in China

*Factors*	*Total* *N (% of 139831)*	*Male* *N (% of 69748)*	*Female* *N (% of 70083)*
Currently smoking*	38943 (27.9)	36544 (52.4)	2399 (3.4)
Frequency of alcohol consumption* ^#^			
No or rarely	109283 (78.1)	42749 (61.3)	66534 (95.0)
Sometimes (< 3 times per week)	18258 (13.1)	15507 (22.2)	2751 (3.9)
Frequently (≥ 3 times per week)	12290 (8.8)	11492 (16.5)	798 (1.1)
Regular exercise in the last 6 months*^&^	19057 (13.6)	9932 (14.2)	9125 (13.0)

* Note: P value < 0.001 for males versus females after adjustment for age, marital status, education, urban/rural residence and geographic region.

^#^P-value < 0.001 for frequent alcohol drinkers versus none, rare or sometimes drinkers.

^&^Regularity of exercise was determined based on respondent's perception.

**Table 6:** Prevalence of smoking, frequent alcohol consumption and physical activity by age and gender in the respondents aged 18 years or older in China

	Male			Female		
		
	Smoking N (%)	Frequently drinking alcohol* N (%)	Regular exercise^#^ N (%)	Smoking N (%)	Frequently drinking alcohol* N (%)	Regular exercise^#^ N (%)
Age						
18–34	8966 (41.8)	1656 (7.7)	2478 (11.5)	218 (1.0)	62 (0.3)	1961 (9.1)
35–44	9504 (60.7)	2902 (18.6)	1525 (9.8)	409 (2.5)	183 (1.1)	1405 (8.7)
45–54	9425 (62.4)	3517 (23.3)	1807 (12.0)	580 (3.9)	230 (1.5)	2015 (13.5)
55–64	4842 (55.4)	1914 (21.9)	1723 (19.7)	464 (5.7)	147 (1.8)	1763 (21.5)
≥ 65	3807 (43.3)	1503 (17.1)	2399 (27.3)	728 (7.9)	176 (1.9)	1981 (21.4)
Residence						
Rural	27245 (54.2)	8736 (17.4)	2789 (5.6)	1615 (3.3)	572 (1.2)	1759 (3.6)
Urban	9299 (47.7)	2756 (14.1)	7143 (36.6)	784 (3.8)	226 (1.1)	7366 (35.5)

* Note: Frequent drinker was defined as drinking ≥ 3 times per week.

^# ^Regularity of exercise was determined based on respondent's perception.

## Discussion

This study highlighted the Chinese adult population health status as the following: 1) only a small proportion of Chinese adults perceived their health as being poor; 2) chronic diseases were high, particularly hypertension, heart disease, chronic pulmonary disease and diabetes; 3) one third of Chinese had a functional problem; 4) prevalence of emotional and/or mental health problems surpassed prevalence of physical functional problems; 5) smoking and alcohol abuse was very common in men; 6) most of the Chinese surveyed were physical inactive and 7) male health status was better than female health status.

Non-communicable, rather than communicable, diseases are the major burden in China and the burden has dramatically increased in the last decade as that in some developing countries [8-10]. Compared to the self-reported health conditions in 1993 [2], our analysis of 2003 national survey data showed that prevalence of hypertension and stroke had doubled and prevalence of diabetes had tripled while the prevalence of pulmonary disease and infectious disease had declined by half. In reality, the prevalence of chronic diseases should be higher than our reports because of unawareness of their presence. For example, we reported prevalence of 3.6% for hypertension and 0.8% for diabetes. Based on previous report of unawareness rate of 55.3% for hypertension [11] and 66.6% for diabetes in China [12], the prevalence should be 8.1% for hypertension and 2.4% for diabetes.

In contrast with the social-economic gradient in health commonly found in the literature [13-16], the wealthier urban population in China is not found to be healthier than the rural population in terms of physician diagnosed chronic disease. Our findings are consistent with a previous report in China [17]. That study measured glucose tolerance among 42,751 residents who were randomly selected from 11 provinces in China, and reported diabetes prevalence rates of 5.8% in municipal areas, 2.9% in high income rural areas and 1.8% in low income rural areas [17].

There are several possible explanations for the above findings. First, the rural population had lower incidence of chronic disease compared with the urban population. The urban population, who has benefited most from China's economic development, has experienced a dramatic lifestyle change in the past two decades. Compared with before, they are becoming more physically inactive (commuting by cars rather than bicycles), and eating more fast food and high protein/fat food. Prior to the implementation of the "open door policy" in the 1980s, China's population health was characterized with a high prevalence of infectious diseases as a result of poverty. It has now shifted to a high prevalence of chronic and non-communicable diseases, brought on by 'affluence.' Such change is much more dominant in urban than rural populations. Of all daily sources of energy, cereals accounted for 61.4% and meat 10.8% for rural residents, compared with 48.5% and 17.6% respectively for urban residents [18].

The second possible explanation is that compared with the urban population, the rural population had a higher mortality rate (6.1/1000 versus 5.6/1000) [19], with a shorter duration from disease occurrence to death, and thus a lower life expectancy (69.5 versus 75.2 years) [20,21]. It was reported that the rural population had higher rates of heart disease and stroke specific mortality than the urban population (330.7 versus 279.5/100,000 person-years for heart disease and 304.1 versus 256.1/100,000 person-years for stroke) [4]. The higher mortality rate is related to lower insurance coverage and lower ability to afford treatment among rural population compared with their counterparts in urban areas [22,23]. China's current healthcare system relies heavily on a non-regulated market to reduce government health expenditure and allows public hospitals to determine the price of services within a certain range [24]. The rural population utilized physicians more than the urban population (52.0% versus 43.0%), but utilized hospitals far less (7.6% versus 11.1%, p < 0.001) when they were ill [25]. More people in rural than in urban areas opted for no treatment when suffering from an illness and were more likely to die earlier.

The third possible explanation is that rural populations were more likely than the urban population to be unaware of the presence of chronic disease. China's national physical measurement study [12] reported that the unawareness of diabetes was 71% and 62% for rural and urban populations, respectively.

Quality of life has been less studied in China although it is an important parameter of population health status. The reason may be due to unawareness of its importance and unavailability of well-developed and validated instruments in the Chinese language. A few previous reports on quality of life either in English or Chinese [26-28] focused on general populations in small geographic areas or patients receiving certain healthcare services. Our study findings of about 30% population with functional problems could not be compared to previous Chinese studies. Compared to a Canadian report [29], our study indicated that Chinese in mainland China had 5% more physical problems but 5% fewer emotional functional problems than Chinese in Canada. However, we have noticed that differences between these two studies in social demographic characteristics and cultural influence were not adjusted.

Health promotion for better nutrition, tobacco and alcohol reduction, increase in exercise, and hypertension control is critical for avoiding population health declining and promote quality of life. Smoking and alcohol abuse was very common in male population, particularly those in the middle age group, but was rare among females. This is related to the Chinese culture, which accepts male smoking and drinking but not female [30]. China's recent health promotion activities have achieved a decline in the male smoking rate from 70% in 1996 to 52% in 2003 [31]. However, many people are still unaware of the dangers in smoking; the proportion who were unaware of smoking's dangers was over 60% in some provinces and higher in rural than urban area [31]. Physical inactivity was common for both men and women. Interestingly, Chinese seniors were more active than the younger populations.

Hypertension is an important risk factor for many chronic diseases, particularly for stroke, heart disease and chronic renal disease. He and colleagues [4] found that hypertension contributed 11.7% to total mortality, smoking 7.9% and physical inactivity 6.8%, resulting in a total of 28.4% (when combined) to mortality in the Chinese adult population. However, these factors were very poorly controlled. About 30% of hypertensive patients took antihypertensive medication with 8.1% achieved blood pressure control [11], and 27.2% of diabetics took medication with 9.7% controlled diabetes [12]. The huge gap between presence, awareness, treatment and control of hypertension strongly indicates imperative needs for a national education program that targets the public, clinicians and decision makers to eliminate the gap. Reforming the healthcare system towards the universal insurance coverage is also essential to remove financial barrier to access the system.

There are four major limitations in the study. First limitation was that validity of self-report health condition was suboptimal. Our prevalence of chronic disease was likely underestimated as stated above. Second limitation was that we did not conduct risk factorial analysis for health status due to the nature of the cross sectional survey. Third limitation is that we did not assess child health status. The reason for that is the survey did not include children under age 15. Fourth limitation is that we only analyzed three major risk factors but were unable to assess other important risk factors of diet and obesity.

## Conclusion

Our analysis demonstrated that males had better health status than females in terms of presence of self-perceived poor wellbeing, illness, chronic disease, and poor quality of life. However, smoking and frequent drinking of alcohol was more prevalent among males than that among females. Our results also indicated that prevalence of chronic illnesses was higher among the urban residents, as compared with rural residents among males and females. Along with reduction of the risk factors to chronic disease, promotion of emotional and mental health should be considered to increase quality of life. Further research on measuring mental health is imperative. Without intervening preventable risk factors for chronic diseases (i.e. reducing hypertension, smoking, alcohol abuse, and physical inactivity), the Chinese population health status will deteriorate even faster as the population ages rapidly due to one-child per family policy in the last thirty years.

## Competing interests

The authors declare that they have no competing interests.

## Authors' contributions

JS designed the study and drafted the manuscript. ML performed the statistical analysis, interpreted the results and participated in coordination. QZ performed the statistical analysis and interpreted the results. MLu participated in the study design and interpretation of the results. HQ conceived the study, participated in its design and drafted the manuscript. All authors read and approved the final manuscript.

## Pre-publication history

The pre-publication history for this paper can be accessed here:



## References

[B1] Cook IG, Dummer TJ (2004). Changing health in China: re-evaluating the epidemiological transition model. Health Policy.

[B2] China Ministry of Health (2006). Health Statistics Yearbook 2006.

[B3] China Ministry of Health Life expectancy.

[B4] He J, Gu D, Wu X, Reynolds K, Duan X, Yao C, Wang J, Chen CS, Chen J, Wildman RP, Klag MJ, Whelton PK (2005). Major causes of death among men and women in China. N Engl J Med.

[B5] China Ministry of Health (2003). The Third National Health Services Survey Design.

[B6] McNutt LA, Wu C, Xue X, Hafner JP (2003). Estimating the relative risk in cohort studies and clinical trials of common outcomes. Am J Epidemiol.

[B7] Spiegelman D, Hertzmark E (2005). Easy SAS calculations for risk or prevalence ratios and differences. Am J Epidemiol.

[B8] Ebrahim S, Smeeth L (2005). Non-communicable diseases in low and middle-income countries: a priority or a distraction?. Int J Epidemiol.

[B9] Lopez AD, Mathers CD, Ezzati M, Jamison DT, Murray CJ (2006). Global and regional burden of disease and risk factors, 2001: systematic analysis of population health data. Lancet.

[B10] Anderson GF, Chu E (2007). Expanding priorities – confronting chronic disease in countries with low income. N Engl J Med.

[B11] Gu D, Reynolds K, Wu X, Chen J, Duan X, Muntner P (2002). Prevalence, awareness, treatment, and control of hypertension in china. Hypertension.

[B12] Wu YF, Xie GQ, Li Y, Zhao LC, Zhou BF (2005). The current status on the prevalence, awareness, treatment and control of diabetes mellitus in several Chinese subpopulations. Chinese J of Epidemioplogy.

[B13] Deaton A (2002). Policy implications of the gradient of health and wealth. Health Aff.

[B14] Wright RJ (2007). Further evidence that the wealthier are healthier: negative life events and asthma-specific quality of life. Thorax.

[B15] McLaren L (2007). Socioeconomic status and obesity. Epidemiol Rev.

[B16] Lorant V, Deliege D, Eaton W, Robert A, Philippot P, Ansseau M (2003). Socioeconomic inequalities in depression: a meta-analysis. Am J Epidemiol.

[B17] Wang KA, Li TL, Xiang DH, Liu ZY, Bai J, Feng JG, Fu ZY, Ma LM, Chen JS, Jin SX, Li YQ, Qian RL, Chen H, Sun TJ, Man QQ (1998). Study on the epidemiological characteristics of diabetes mellitus and IGT in China. Chinese J of Epidemiology.

[B18] Li LM, Rao KQ, Kong LZ, Yao CH, Xiang HD, Zhai FY, Ma GS, Yang XG (2005). A description on the Chinese national nutrition and health survey in 2002. Chinese J of Epidemiology.

[B19] Ministry of Health, China (2004). China Health Statistics Yearbook.

[B20] China Statistics Department (2006). Statistics of 1% national sample, 2005.

[B21] Information Centre, Helath Ministry of China (2003). China health development report, 1997–2001.

[B22] Jing F (2004). Health sector reform and reproductive health services in poor rural China. Health Policy Plan.

[B23] Blumenthal D, Hsiao W (2005). Privatization and its discontents – the evolving Chinese health care system. N Engl J Med.

[B24] Ma J, Lu M, Quan H (2008). Healthcare system from central planning to market-based: lessons from China. Health Aff.

[B25] Liu M, Zhang Q, Lu M, Kwon CS, Quan H (2007). Rural and urban disparity in health services utilization in China. Med Care.

[B26] Liang XY, Nie SF, Qu KY, Peng XX, Wei S, Zhu GB, Wu LJ, Guo XH, Xiao R, Ju LR, Wang W (2006). Evaluation of health-related quality of life among hypertensive patients in a rural area, PR China. J Hum Hypertens.

[B27] Tang WL, Wang YM, Du WM, Cheng NN, Chen BY (2006). Assessment of quality of life and relevant factors in elderly diabetic patients in the Shanghai community. Pharmacoepidemiol Drug Saf.

[B28] Xu J, Wang M, Xiang Y, Hu X (2005). Quality of life for people with intellectual disabilities in China: a cross-culture perspectives study. J Intellect Disabil Res.

[B29] Leung B, Lu N, So L, Quan H (2007). Comparing three measures of health status (perceived health with Likert-type scale, EQ-5D, and number of chronic conditions) in Chinese and White Canadians. Med Care.

[B30] Ma GS, Sun LZ, Luan DC, Li YP, Hu XQ, Wang JZ, Yang XG (2005). The description analysis of the smoking pattern of people in China. Chinese J of Prevention and Control for Chronic Disease.

[B31] Yang GH, Ma JM, Liu N, Zhou LN (2005). Smoking and passive smoking in Chinese, 2002. Chinese J of Epidemiology.

